# Circulating Soluble TREM2 and Cardiovascular Outcome in Cohort Study of Coronary Atherosclerosis Patients

**DOI:** 10.3390/ijms232113121

**Published:** 2022-10-28

**Authors:** Valeri Cuciuc, Sagi Tshori, Livi Grib, Gal Sella, Ortal Tuvali, Igor Volodarsky, Michael Welt, Michael Fassler, Sara Shimoni, Jacob George

**Affiliations:** 1 Heart Center, Kaplan Medical Center, Faculty of Medicine, The Hebrew University of Jerusalem, Rehovot 7661041, Israel; 2 Research Authority, Kaplan Medical Center, Faculty of Medicine, The Hebrew University of Jerusalem, Rehovot 7661041, Israel; 3 Department of Cardiology, Nicolae Testemitanu State University of Medicine and Pharmacy, 2004 Chișinău, Moldova

**Keywords:** Triggering Receptor Expressed in Myeloid Cells 2, sTREM2, atherosclerosis, death, inflammation, ischemic heart disease

## Abstract

Triggering Receptor Expressed in Myeloid Cells 2 (TREM2) is a membrane receptor in myeloid cells that mediates cellular phagocytosis and inflammation. TREM2 and its soluble extracellular domain are clearly implicated in neuroinflammation and neurodegeneration. sTREM2 is also expressed in atherosclerotic macrophages. We hypothesized that sTREM2 would predict cardiovascular mortality in patients with established coronary atherosclerosis (CAD). Consecutive patients undergoing coronary angiography with the establishment of the diagnosis of CAD (*n* = 230) and without CAD (*n* = 53) were tested for their baseline serum sTREM2 levels. All patients were followed up for 84 months or until death occurred. sTREM2 correlated with age; however, no association was found between sTREM2 and the number of atherosclerotic vessels involved (*p* = 0.642). After 84 months of follow-up, 68 out of the 230 CAD patients had died. After adjusting for age and other risk factors, the adjusted hazard ratio for the highest quartile of sTREM2 was 2.37 (95% confidence interval 1.17–4.83) for death. In patients with established CAD, serum sTREM2 appears to predict cardiovascular death as a potential surrogate for plaque rupture. TREM2 and its soluble extracellular form might be implicated in the fate of the atherosclerotic plaque, but corroboration within larger studies is needed.

## 1. Introduction

Triggering Receptor Expressed in Myeloid Cells 2 (TREM2) is a pattern-recognition receptor, a member of the immunoglobulin superfamily that is expressed on myeloid cells [1]. TREM2 is expressed by macrophages, dendritic cells, and osteoclasts in the periphery [2,3,4,5]. In the central nervous system, the expression of TREM2 is confined to microglia, and it is one of the more highly expressed molecules in microglia [6,7]. TREM2 engagement results in intracellular signaling mediated by its association with TYRO protein tyrosine kinase-binding protein (TYROBP), also known as DNAX-activating protein of 12 kDa (DAP12) [3,6,8]. A variety of ligands were shown to be associated with TREM2, including anionic lipids, apolipoprotein E, Lipopolysaccharide (LPS), bacteria, etc. TREM2 appears to play a central role in myeloid functions, including apoptosis, uptake/phagocytosis of anionic lipids/debris/bacteria, and inflammation [1]. Much of the recent interest in TREM2 follows the observation that genome-wide association studies (GWAS studies) have suggested that its mutations are associated with a higher incidence of neurodegenerative diseases such as Alzheimer’s [9,10]. These observations have fueled research aiming to explore the role of neuroinflammation in the pathogenesis of neurodegeneration.

TREM2 comprises an extracellular domain that mediates ligand binding and is either alternatively spliced or shed via the proteases A Disintegrin and Metalloproteinase Domain 10 (ADAM10) and ADAM17 [11]. The levels of soluble forms of TREM2 appear to mirror the extent of local inflammation. Thus, when microglia cells are activated, higher sTREM2 levels are observed in early Alzheimer’s disease in the cerebrospinal fluid, and these appear to predict the worsening of dementia in these patients [11].

Atherosclerosis is associated with continuous activation of the innate and adaptive immune system [12]. Macrophages, the principal innate immune cells in atherosclerotic plaques that have been shown to express TREM2, are the equivalent cellular element that parallel microglia in the central nervous system in the context of removing toxic debris, controlling innate immunity, and dictating plaque composition.

Single-cell RNA-sequencing studies have demonstrated that TREM2-expressing macrophages are an abundant macrophage subtype in murine atherosclerotic lesions and that the majority of the foamy macrophages in atherosclerotic lesions express high levels of TREM2 [13,14,15] that can also potentially influence their functional properties [16]. A higher expression of membrane-bound TREM2 in plaque-associated macrophages in vulnerable lesions is likely to result in increased concentrations of soluble TREM2 as there is a direct association between the bound and shed protein. No study has yet tested the levels of circulating soluble TREM2 in patients with coronary atherosclerosis.

In this study, we aim to test the hypothesis that levels of circulating sTREM2 can serve as a biomarker of atherosclerotic plaque activity by associating sTREM2 levels prospectively with cardiovascular outcome in patients with coronary atherosclerosis.

## 2. Results

A total of 283 patients were included in the final cohort, including 230 patients with significant coronary atherosclerosis (CAD) and 53 patients without significant atherosclerosis and without prior ischemic history (non-CAD). The mean age was 67 ± 12 years and 66% were males in the CAD group, similarly to the non-CAD group (Table 1). There was a slightly higher prevalence of diabetes mellitus, essential hypertension, and hyperlipidemia in the CAD group. sTREM2 levels were similar in the CAD and non-CAD groups (9115 ± 5969 pg/mL vs. 8049 ± 4518 pg/mL, respectively).

**Figure 1:**
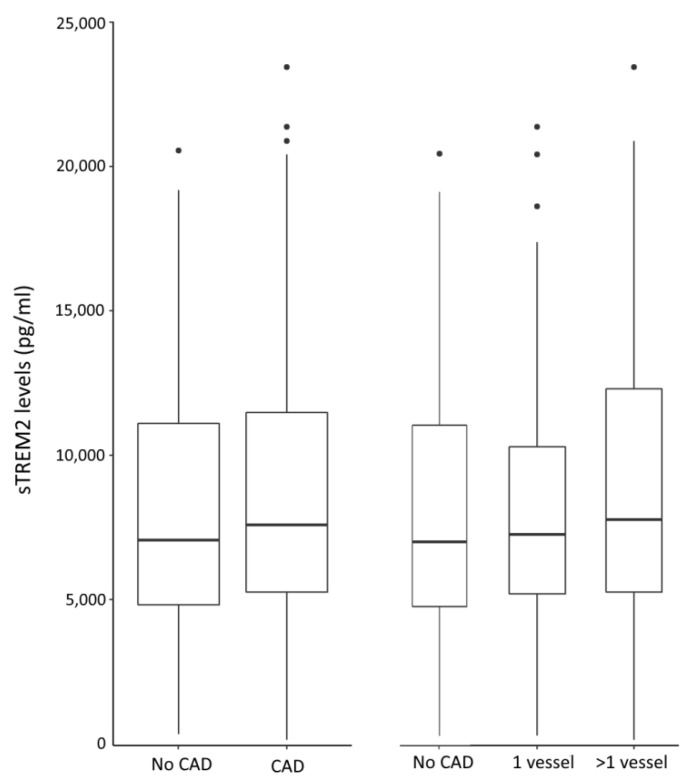
Circulating soluble TREM2 was quantified by performing ELISA in patient sera. sTREM2 levels are depicted according to the coronary atherosclerosis status and the number of involved vessels. Individual outliers are shown. No CAD- no current or previous coronary atherosclerosis.

sTREM2 levels were compared in the CAD vs. non-CAD groups and in patients with single-vessel or multi-vessel disease (Figure 1). No significant changes in sTREM2 levels were noted in either CAD vs. non-CAD patients (*p* = 0.642), non-CAD patients vs. single- and multi-vessel disease (*p* = 0.806), or single- vs. multi-vessel disease (*p* = 0.720).

**Figure 2:**
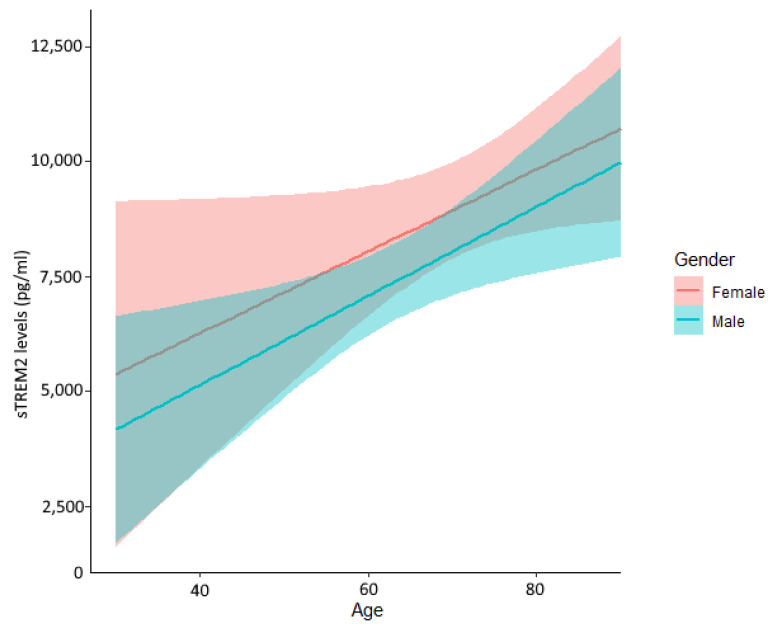
Circulating soluble TREM2 levels stratified by age and sex. All patients were included. sTREM2 level is associated with age in both males and females.

Linear regression analysis adjusted by age, sex, and clinical risk factors showed no significant association was found with either sex or cardiac risk factors (Table 2). sTREM2 levels were significantly associated with age (β = 0.008 [95% CI 0.005–0.010] per year for log sTREM2, *p* < 0.001). Serum sTREM2 levels increased with age in both males and females (Figure 2).

**Figure 3:**
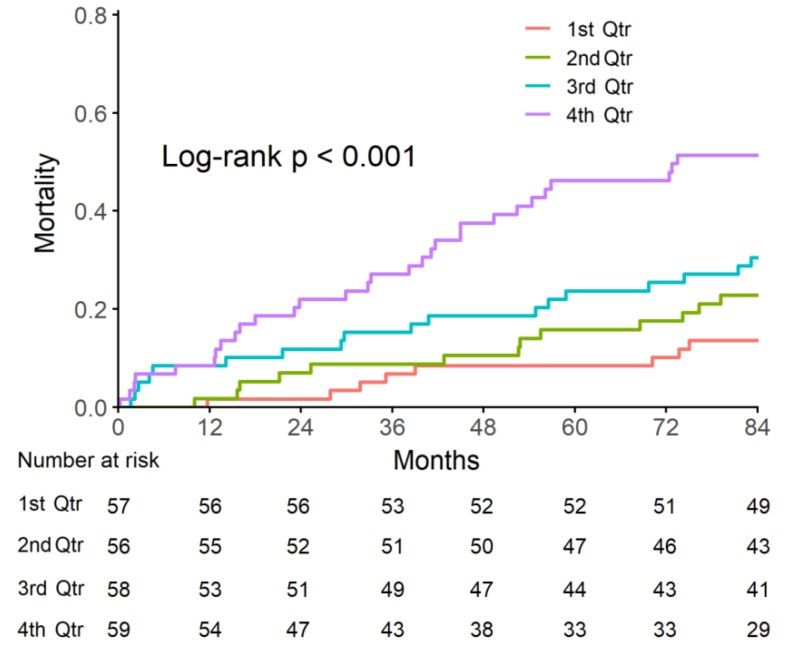
sTREM2 stratified by quartiles and the Risk of Death. Mortality was significantly higher in the higher quartiles of sTREM2. The number at risk over time in each group is indicated.

Because serum sTREM2 levels are not associated with either the extent of coronary atherosclerosis or cardiac risk factors, we assessed if sTREM2 is an independent predictor of mortality in CAD patients. During our follow-up, 81 deaths were observed. sTREM2 levels were categorized into quartiles. In the lowest sTREM2 quartile group, eight patients died after seven years (14% mortality). In contrast, 30 patients in the highest quartile group died (51% mortality). The Kaplan–Meier curve indicates that higher sTREM2 levels were significantly associated with a 7-year mortality rate (Figure 3).

Cox regression analysis showed that sTREM2 level was associated with a significantly higher mortality in both the unadjusted and adjusted models (Table 3). Mortality was significantly associated with age and was borderline associated with atrial fibrillation. The multivariable-adjusted hazard ratio for death for the highest quartile of sTREM2 was 2.37 (95% CI 1.17–4.83, *p* = 0.016).

**Figure 4:**
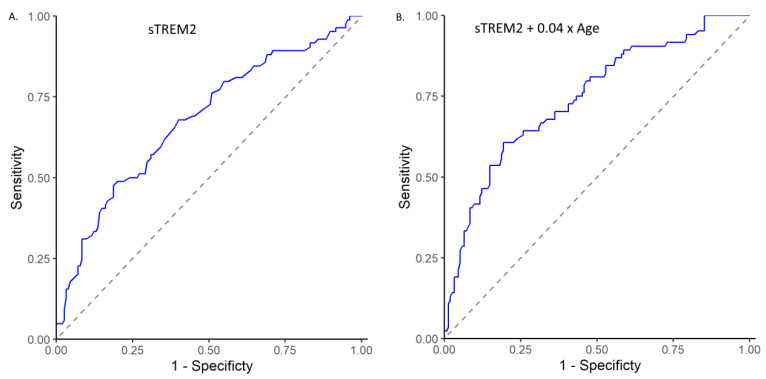
ROC curve representing area under the curve, sensitivity, and specificity for mortality based on log sTREM2 levels alone (**A**) or on linear combination of sTREM2 with age (**B**).

We also assessed the ability of sTREM2 levels to predict mortality using receiver operating characteristic curves (ROC curves) (Figure 4). Serum log sTREM2 levels achieved an area under the curve (AUC) of 0.679 (95% CI 0.605–0.752) indicating a useful discrimination for patients with a higher risk of mortality. We also used a second predictive model with a linear combination of log sTREM2 levels and age. The optimal model, log sTREM2 + 0.04 × age, achieved an AUC of 0.745 (95% CI 0.677–0.814), indicating a better discrimination for patients with a higher risk of mortality.

## 3. Discussion

Atherosclerosis is a chronic progressive process, and data support the involvement of innate and adaptive immune responses in its pathogenesis [12]. In this respect, macrophages that constitute the central cellular element within the atherosclerotic plaque are also principal innate immune effectors of the process [12].

There are recent data on the expression of TREM2 in various types of macrophages, such as tumor-associated macrophages (TAMs) and liver macrophages [17]. Recent gene-profiling study data also suggest that TREM2 expression is abundant in atherosclerotic plaques, although their precise role is yet to be delineated [13,14].

As the peripheral blood serves as the window for the vasculature, we reasoned that if the number of activated vessel-wall macrophages increases, this would signal a plaque that is more likely to be inflamed and thus associated with future rupture and a consequent worsening of cardiovascular outcome.

The first observation from this study is that we could not detect a statistically significant difference between the extent of atherosclerosis assessed by the baseline coronary angiography and the levels of sTREM2. This finding can be related to the relatively small size of this hypothesis-generating study or to the limitation inherent in assessing the extent of atherosclerosis by coronary angiography. However, we did observe that circulating soluble TREM2 concentrations were significantly associated with age. As age is a continuous determinant that is associated with atherosclerosis, this may suggest that if atherosclerosis can be accurately measured throughout the vascular system, the association with circulating sTREM2 may become statistically significant. Additionally, it is well accepted that aging is associated with a heightened global inflammatory response consistent with the association we detected between age and soluble TREM2.

The most important finding of this long prospective follow up study of 84 months is the association of baseline circulating sTREM2 with cardiovascular mortality. This was gradual across sTREM2 quartiles and particularly evident in the upper quartile where curves continued to separate as time elapsed from the baseline assessment.

Cardiovascular mortality in patients with proven preexisting evidence of coronary atherosclerosis may primarily be related to the occurrence of myocardial infarctions as a result of plaque rupture/erosion. Thus, we hypothesize that a heightened innate immune activation mirrored by increased circulating soluble TREM2 shed from plaque macrophages results in increased cardiovascular mortality, likely primarily related to plaque destabilization and subsequent rupture. Interestingly, a very recent study demonstrated circulating soluble TREM2 to be associated with death and cardiovascular events in patients with ischemic stroke, a population with a high pretest probability of concomitant coronary atherosclerosis [18].

There have been many studies aiming to study biomarkers that may predict outcomes in patients with atherosclerosis. A biomarker that further improves outcome prediction beyond LDL cholesterol levels will help in the selection of patient subgroups that can be more intensively monitored and treated [19]. CRP is a highly tested biomarker with data supporting its predictive power on outcome, yet it has not entered routine testing as it did not attain a sufficiently robust guideline endorsement. The advantage of soluble TREM2 as compared to other biomarkers, principally inflammatory in nature, is the more specific attributes of this biomarker. Whereas markers like CRP, IL-6, IL-2, etc., can be produced by various inflammatory or endothelial cells in the circulation and atherosclerotic plaques, TREM2 is not produced by circulating peripheral mononuclear cells but rather only by macrophages in the plaques. When macrophages are activated, the expression of TREM2 increases with subsequent shedding and may thus stand as a more specific biomarker of plaque destabilization.

An intriguing point raised by this hypothesis-generating study relates to the potential role of the extracellular shed soluble TREM2 to act not just as a biomarker but also as a potential facilitator of plaque rupture. We and others have recently shown that injection of soluble TREM2 to murine brains triggers neuroinflammation and is thus capable of activating immune cell subpopulations [20,21]. It is not yet known if this finding is related to the ability of soluble TREM2 to act as a decoy receptor or alternatively to agonize a yet-unidentified cognate receptor [11]. Regardless of the mechanism, it is intriguing to speculate that soluble TREM2 may shape the composition and behavior of the atherosclerotic plaque, especially in view of the therapeutic agents targeting TREM2 that are currently under early clinical testing [22].

### Study Limitations

The main limitation of this research is that it is a single-center study with a relatively small study cohort. This also limited our ability to demonstrate the incremental value of sTREM2 in outcome prediction in addition to known predictors such as Troponin. Another limitation is that we did not substantiate the mechanism with data from macrophage experiments but rather mainly by the effect of sTREM2 on various macrophages subsets.

## 4. Materials and Methods

### 4.1. Study Population

This study included 311 patients who underwent coronary angiography in the Heart Center of Kaplan Medical Center from 2010 to 2013. The study was approved by the Kaplan Medical Center Institutional Review Board (0109-10-KMC, 20 September 2010). Patients provided their informed consent and had serum collected close to the time of the procedure. We collected clinical data including demography, comorbidities, medications, and laboratory data. Mortality follow-up was based on the patients’ electronic health records. Patients were followed up until the end of 2019 or death. Due to missing data, 18 patients were removed from analysis, and 10 patients were removed from the non-CAD group due to previous ischemic history. Atherosclerotic stenosis >70% was classified as significant coronary atherosclerosis. The final study population included 283 patients, 230 of whom had at least one vessel with stenosis of ≥70% (65 patients with single-vessel disease and 165 with multi-vessel disease), and 53 patients without significant coronary atherosclerosis.

### 4.2. Determination of sTREM2 Levels in Serum

The soluble TREM2 (sTREM2) level was determined in the collected sera using a Human sTREM2/Triggering Receptor Expressed on Myeloid Cells 2 ELISA Kit (Sunlong Biotech, HangZhou, China) according to the manufacturer’s instructions. In brief, serum samples were diluted with sample dilution buffer. Samples were incubated at 37 °C for one hour, followed by washing. A Horseradish Peroxidase (HRP)-conjugated antibody specific for sTREM2 was added to each microplate well. Samples were incubated at 37 °C for one hour, followed by washing. A TMB substrate solution was added. The enzyme-substrate reaction was terminated by the addition of a sulfuric acid solution, and the color change was measured spectrophotometrically at a wavelength of 450 nm ± 2 nm. The concentration of sTREM2 in the samples was determined by comparing the O.D. of the samples to the O.D. of a standard curve in the same ELISA plate.

### 4.3. Statistical Methods

The effect of coronary atherosclerosis on sTREM2 was calculated with either the χ2 test with sTREM2 levels as a categorical variable using quartiles as groups or an independent *t*-test using sTREM2 levels. The factors associated with sTREM2 level were assessed using univariate and multivariate linear regression. All mortality analyses were performed on the subgroup of coronary atherosclerosis patients. We used Kaplan–Meier cumulative incidence curves to assess the effect of sTREM2 levels on mortality with a log-rank test. Univariate and multivariate Cox proportional hazards regression models were also used. sTREM2 levels, age, sex, BMI, diabetes, dyslipidemia, hypertension, smoking status, atrial fibrillation, family history of acute coronary disease, and the presence of single- or multi-vessel disease were introduced in the adjusted model. Both the crude hazard ratio (HR) and adjusted HR (aHR) are presented with 95% confidence intervals. To further evaluate the predictive value of sTREM2, we performed a receiver operating characteristic (ROC) curve analysis on the mortality rate based on sTREM2 levels alone or in combination with age, and the formula with optimal AUC is presented. Optimal cutoff values were calculated using Youden’s index. Statistical analysis was carried out using IBM SPSS version 25 and R version 4.0.3 with the survival, survminer, and ROCit packages.

## 5. Conclusions

This is the first study to show that circulating soluble TREM2 levels can independently predict long-term cardiovascular outcomes in patients with coronary atherosclerosis. If this finding is further corroborated by larger studies, soluble TREM2 may stand as a novel biomarker of prognosis in patients with coronary atherosclerosis and pave the way to an improved understanding of the mechanisms governing plaque destabilization.

## Figures and Tables

**Table 1:** Baseline characteristic of CAD and non-CAD cohorts and serum soluble TREM2 levels.

	CAD (*n* = 230)	Non-CAD (*n* = 53)
sTREM2 (pg/mL)	9115 ± 5969	8049± 4518
Age (years)	67 ± 12	65 ± 14
Male	152 (66%)	33 (63%)
BMI (kg/m^2^)	28.9 ± 5.4	28.1 ± 6.0
Diabetes mellitus	92 (40.0%)	18 (34.0%)
Hypertension	172 (74.8%)	34 (64.2%)
Hyperlipidemia	158 (68.7%)	26 (49.1%)
Smoking (current)	42 (18.3%)	10 (18.9%)
PVD	10 (4.4%)	1 (1.8%)
CVA	19 (8.3%)	3 (5.7%)
Chronic renal failure	44 (19.1%)	8 (15.1%)

Baseline characteristics classified according to significant CAD (>70% stenosis on coronary angiography). PVD, peripheral vascular disease; CVA, cerebrovascular accident; BMI, Body mass index; sTREM2, soluble TREM2. Values are expressed as either number (%) or mean ± SD.

**Table 2:** Patient Characteristics Associated With soluble TREM2 Concentration.

	Unadjusted Model			Adjusted Model		
	β1	95% CI	*p*-Value	β1	95% CI	*p*-Value
Age (years)	0.009	0.007–0.011	<0.001	0.008	0.005–0.010	<0.001
Sex	0.124	0.063–0.185	<0.001	−0.056	−0.122	0.132
Diabetes mellitus	0.095	0.036–0.155	0.008	0.039	−0.118	0.274
Hypertension	0.18	0.116–0.244	<0.001	0.076	0.007–0.144	0.068
Past smoking	−0.043	−0.172	0.411	0.087	0.003–0.170	0.089
Current smoking	−0.096	−0.174, −0.018	0.044	0.06	−0.167	0.236
PVD	0.115	−0.294	0.197	0.055	−0.293	0.532
Atherosclerosis	0.038	−0.144	0.388	0.033	−0.134	0.422

PVD, peripheral vascular disease.

**Table 3:** Crude and adjusted HRs for mortality by sTREM2 and risk factors.

	Unadjusted Model		Adjusted Model	
	HR (95% CI)	*p*-Value	Adjusted HR (95% CI)	*p*-Value
log sTREM2	7.050 (2.943–16.891)	<0.001	3.857 (1.419–10.485)	0.008
Age	1.061 (1.038–1.083)	<0.001	1.052 (1.023–1.081)	<0.001
Sex	1.315 (0.841–2.055)	0.230	0.924 (0.563–1.518)	0.756
BMI (kg/m^2^)	1.018 (0.977–1.060)	0.394	1.017 (0.975–1.061)	0.428
Diabetes	1.302 (0.891–1.903)	0.173	1.089 (0.697–1.700)	0.709
HTN	1.762 (0.991–3.133)	0.054	0.881 (0.469–1.656)	0.694
Smoking	0.595 (0.356–0.994)	0.047	1.096 (0.609–1.972)	0.760
AF	3.277 (2.051–5.233)	<0.001	1.647 (0.987–2.750)	0.056
Dyslipidemia	1.276 (0.782–2.081)	0.329	0.942 (0.559–1.589)	0.824
Family history	0.736 (0.268–2.019)	0.552	1.656 (0.563–4.869)	0.360
Single- vs. multi-vessel	0.639 (0.406–1.005)	0.053	0.714 (0.418–1.218)	0.216

HTN, essential hypertension; AF, atrial fibrillation; Single-vessel, single-vessel disease with obstruction > 70%.

## Data Availability

Not applicable.
